# Supplementary material for: The Extent of Lifestyle-Induced Weight Loss Determines the Risk of Prediabetes and Metabolic Syndrome Recurrence during a 5-Year Follow-Up
Source: Nutrients. 2022 Jul 26;14(15):3060. doi: 10.3390/nu14153060 (PMC9331424; doi:10.3390/nu14153060)
Supplement: Supplementary file 1 [file nutrients-14-03060-s001.zip › nutrients-1813425-supplementary.pdf]

# The extent of lifestyle-induced weight loss determines the risk of prediabetes and metabolic syndrome recurrence during a 5-year follow-up

**Silke Zimmermann [1*], Mandy Vogel [2], Akash Mathew [1], Thomas Ebert [3,4], Rajiv Rana [1], Shihai Jiang [1], Berend Isermann [1] and Ronald Biemann [1*]**

[1] Institute of Laboratory Medicine, Clinical Chemistry and Molecular Diagnostics, University of Leipzig, 04103 Leipzig, Germany
[2] Hospital for Children and Adolescents, Centre for Pediatric Research, Department Woman and Child Health, University of Leipzig, Leipzig, Germany
[3] Karolinska Institutet, Department of Clinical Science, Intervention and Technology, Division of Renal Medicine, SE-141 86 Stockholm, Sweden
[4] Medical Department III – Endocrinology, Nephrology, Rheumatology, University of Leipzig Medical Center, Leipzig, Germany

*Correspondence: ronald.biemann@medizin.uni-leipzig.de (RB); silke.zimmermann@medizin.uni-leipzig.de (SZ)

## Supplementary Materials:

**Supplementary Table S1: Clinical parameters of individuals with metabolic syndrome at baseline and after the 6-month lifestyle-induced weight loss intervention period.** Data are presented as median (interquartile range). Wilcoxon Signed-Rank Test was used to analyze differences of paired samples between baseline (BL) and lifestyle-induced weight loss in each quartile (Q1-Q4).

| Parameters | BL | Q1 | *p* | BL | Q2 | *p* | BL | Q3 | *p* | BL | Q4 | *p* |
|---|---|---|---|---|---|---|---|---|---|---|---|---|
| **Age [years]** | 47.5 ± 9 | | | 48 ± 6 | | | 49 ± 5 | | | 47.5 ± 10.3 | | |
| **BMI** | 32.9 ± 6.6 | 30.9 ± 7.3 | <0.01 | 33.0 ± 5.7 | 28.8 ± 4.9 | <0.01 | 35.4 ± 3.4 | 30.0 ± 3.5 | <0.01 | 35.2 ± 3.2 | 27.5 ± 5.1 | <0.01 |
| **Weight [kg]** | 106.2 ± 28 | 100.1 ± 27.6 | <0.01 | 107 ± 21.8 | 94.5 ± 18.2 | <0.01 | 108 ± 15.9 | 93.5 ± 14.5 | <0.01 | 109.8 ± 20 | 12.7 ± 15.3 | <0.01 |
| **HDL cholesterol [mmol/L]** | 1.41 ± 0.7 | 1.53 ± 0.8 | n.s. | 1.2 ± 0.4 | 1.33 ± 0.4 | <0.01 | 1.23 ± 0.4 | 1.45 ± 0.6 | <0.01 | 1.46 ± 0.4 | 1.46 ± 0.4 | <0.01 |
| **TG [mmol/L]** | 2.19 ± 2.2 | 2.16 ± 2.8 | n.s. | 2.0 ± 2.6 | 1.36 ± 0.8 | <0.01 | 2.03 ± 1.9 | 1.4 ± 0.8 | <0.01 | 1.83 ± 1.5 | 0.98 ± 0.4 | <0.01 |
| **$RR_{sys}$ [mmHg]** | 140 ± 12 | 138 ± 16.0 | n.s. | 138 ± 14.0 | 122 ± 13.0 | <0.01 | 140 ± 28.5 | 130 ± 14.0 | n.s. | 149.0 ± 18.0 | 130 ± 18.0 | 0.02 |
| **HbA1c [%]** | 5.6 ± 0.5 | 5.5 ± 0.4 | n.s. | 5.6 ± 0.7 | 5.4 ± 0.3 | n.s. | 5.4 ± 0.8 | 5.3 ± 0.7 | n.s. | 5.6 ± 0.5 | 5.2 ± 0.6 | 0.02 |
| **Insulin [pmol/L]** | 88 ± 80 | 68 ± 28 | <0.01 | 79.5 ± 72.3 | 45.5 ± 24 | <0.01 | 68 ± 83.3 | 36 ± 43.3 | <0.01 | 60 ± 52 | 30 ± 21 | <0.01 |
| **Glucose [mmol/L]** | 5.9 ± 0.8 | 5.8 ± 0.7 | n.s. | 6.2 ± 1.2 | 5.7 ± 0.6 | 0.02 | 5.9 ± 1.2 | 5.4 ± 0.6 | n.s. | 6.2 ± 0.9 | 5.6 ± 0.7 | 0.02 |

**Supplementary Table S2.** Table showing the number of participants treated with antilipidemic drugs and total numbers of participants in each quartile (Q1-Q4) at each timepoint. Fisher's exact probability test was used to analyze differences in the number of treated versus nontreated participants at different timepoints (only significant values are listed). BL= baseline; LIWL= lifestyle-induced weight loss; FU1-5= years of follow-up

| | **Q1 antilipidemics/ total** | **Q2 antilipidemics/ total** | **Q3 antilipidemics/ total** | **Q4 antilipidemics/ total** | ***p*** |
|---|---|---|---|---|---|
| BL | 4/15 | 5/14 | 3/14 | 3/15 | n.s. |
| FU1 | 4/11 | 4/12 | 4/11 | 4/14 | n.s. |
| FU2 | 4/8 | 4/11 | 4/11 | 4/12 | n.s. |
| FU3 | 4/8 | 4/8 | 5/10 | 4/11 | n.s. |
| FU4 | 5/6 | 4/10 | 4/11 | 4/12 | n.s. |
| FU5 | 4/7 | 4/9 | 4/11 | 4/13 | n.s. |

**Supplementary Table S3.** Median and interquartile ranges of $RR_{dia}$ levels before and after LIWL and during the 5-year follow-up. The Wilcoxon signed rank test was used to analyze changes between paired samples between BL and subsequent follow-up timepoints (only significant values are listed). BL= baseline; LIWL= lifestyle-induced weight loss; FU1-5= years of follow-up.

| **$RR_{dia}$ (mmHg)** | **BL** | **Q1** | ***p*** | **BL** | **Q2** | ***p*** | **BL** | **Q3** | ***p*** | **BL** | **Q4** | ***p*** |
|---|---|---|---|---|---|---|---|---|---|---|---|---|
| **LIWL** | 90 ± 12 | 90 ± 10 | n.s. | 84 ± 15 | 81 ± 7.3 | n.s. | 85 ± 13 | 84.5 ± 8 | n.s. | 91 ± 15.5 | 84.5 ± 9.5 | n.s. |
| **FU1** | | 90 ± 23 | n.s. | | 84 ± 12 | n.s. | | 82 ± 11 | n.s. | | 84 ± 12 | n.s. |
| **FU2** | | 99 ± 30.8 | n.s. | | 85 ± 17.3 | n.s. | | 90 ± 13.5 | n.s. | | 82 ± 15.5 | n.s. |
| **FU3** | | 88 ± 16 | n.s. | | 88 ± 12 | n.s. | | 85 ± 17.5 | n.s. | | 86 ± 9 | n.s. |
| **FU4** | | 88 ± 21 | n.s. | | 85 ± 18 | n.s. | | 90 ± 18.3 | n.s. | | 86 ± 19 | n.s. |
| **FU5** | | 85 ± 8 | n.s. | | 89 ± 11.8 | n.s. | | 85 ± 10 | n.s. | | 81 ± 11.8 | <0.01 |

**Supplementary Table S4.** Table showing the number of participants treated with antihypertensive medication (AHM) and total numbers of participants in each quartile (Q1-Q4) at each timepoint. Fisher's exact probability test was used to analyze differences in the number of treated versus nontreated participants at different timepoints (only significant values are listed). BL= baseline; LIWL= lifestyle-induced weight loss; FU1-5= years of follow-up

| | **Q1 AHM/ total** | **Q2 AHM/ total** | **Q3 AHM/ total** | **Q4 AHM/ total** | ***p*** |
|---|---|---|---|---|---|
| BL | 11/15 | 11/14 | 13/14 | 12/15 | n.s. |
| FU1 | 10/11 | 9/12 | 7/11 | 6/14 | n.s. |
| FU2 | 5/8 | 10/11 | 9/11 | 6/12 | n.s. |
| FU3 | 8/8 | 8/8 | 9/10 | 6/11 | 0.02 |
| FU4 | 5/6 | 10/10 | 10/11 | 8/12 | n.s. |
| FU5 | 6/7 | 9/9 | 10/11 | 7/13 | 0.04 |

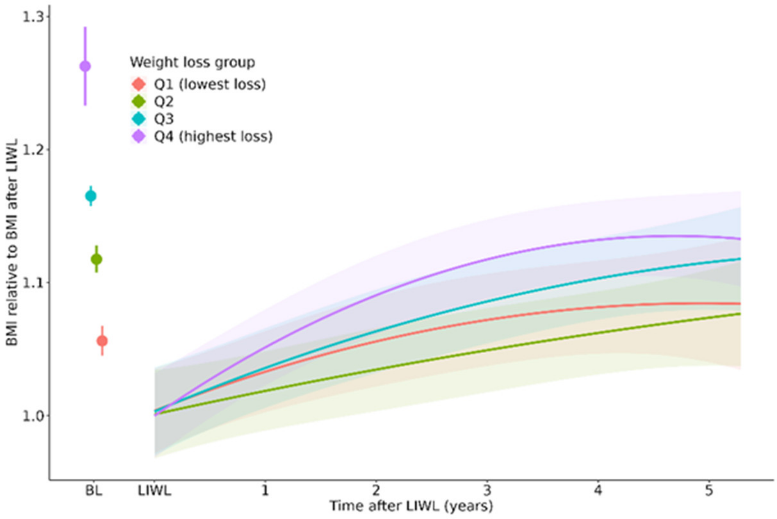


**Supplementary Figure S1.** BMI trends relative to BMI after lifestyle-induced weight loss in weight loss stratified by quartiles Q1-Q4 in a modeled quadratic dependent on time after LIWL.Data are presented as change in BMI since LIWL. BL= baseline; LIWL= lifestyle-induced weight loss; 1-5= years of follow-up

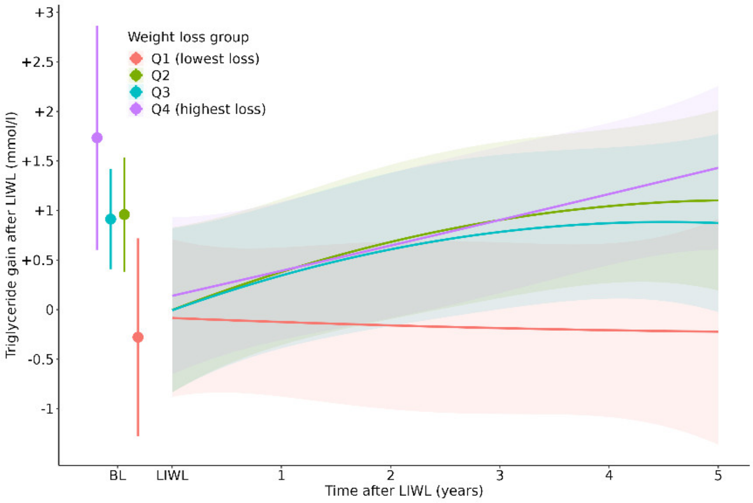


**Supplementary Figure S2.** Triglyceride level trends relative to triglyceride levels after lifestyle-induced weight loss stratified by weight loss quartiles Q1-Q4 in a modeled quadratic dependent on time after LIWL. Data are presented as change in Triglycerides after LIWL. BL= baseline; LIWL= lifestyle-induced weight loss; 1-5= years of follow-up

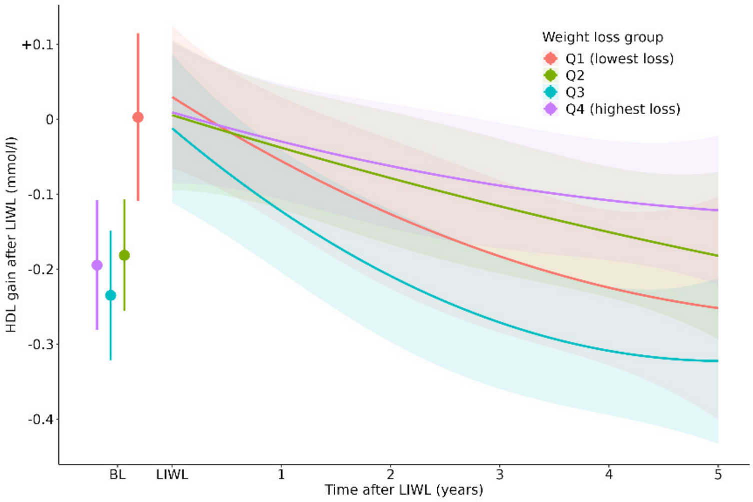


**Supplementary Figure S3.** HDL cholesterol relative to HDL cholesterol after lifestyle-induced weight loss stratified by weight loss quartiles Q1-Q4 in a modeled quadratic dependent on time after LIWL. Data are presented as change in HDL cholesterol after LIWL. BL= baseline; LIWL= lifestyle-induced weight loss; 1-5= years of follow-up

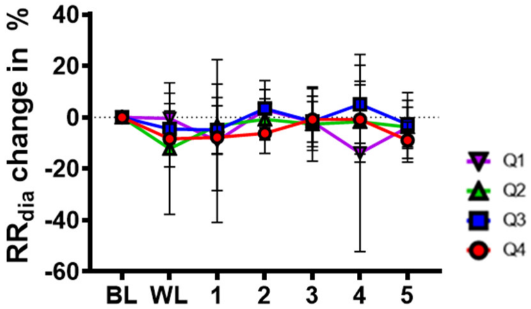


**Supplementary Figure S4:** Long-term effects of lifestyle-induced weight loss on diastolic blood pressure during the 5-year follow-up. Data are presented as the median (interquartile range). BL= baseline; WL= weight loss; 1-5= years of follow-up.

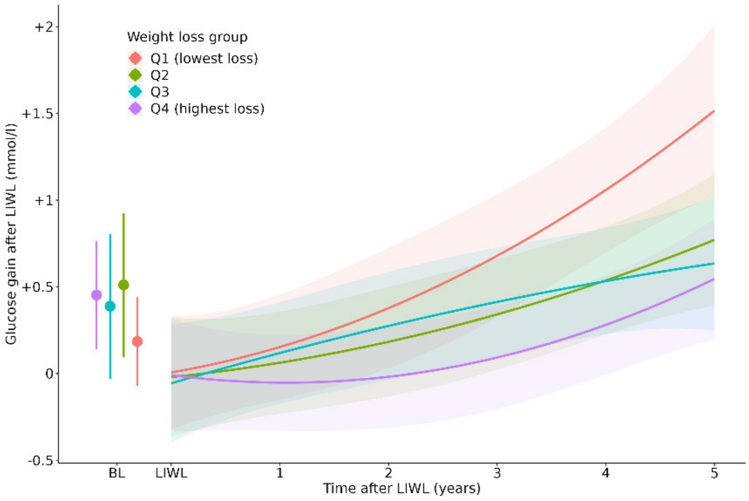


**Supplementary Figure S5.** Fasting plasma glucose relative to fasting plasma glucose after lifestyle-induced weight loss stratified by weight loss quartiles Q1-

Q4 in a modeled quadratic dependent on time after LIWL. Data are presented as change in fasting plasma glucose after LIWL. BL= baseline; LIWL= lifestyle-induced weight loss; 1-5= years of follow-up
